# Treating TNF Receptor Associated Periodic Fever Syndrome in End-Stage Renal Failure

**DOI:** 10.1155/2019/6819476

**Published:** 2019-03-17

**Authors:** J. Coutinho, R. S. Chorão, M. Oliveira, C. R. Santos

**Affiliations:** ^1^Hospital Amato Lusitano, Castelo Branco, Portugal; ^2^Centro Hospitalar Cova da Beira, Covilhã, Portugal

## Abstract

Tumor necrosis factor receptor associated periodic syndrome (TRAPS) is a rare monogenic autoinflammatory disease. Its most severe manifestation is secondary amyloidosis. A 44-year-old male presented with nephrotic syndrome. Kidney biopsy was conclusive for secondary amyloidosis. The patient and his children had a history of recurrent febrile periods since infancy. All subjects were positive for a heterozygous variant of the TNFRSF1A gene, confirming TRAPS diagnosis. The patient progressed to end-stage renal failure and developed recurrent pericarditis episodes. He was started on anakinra while on hemodialysis with marked reduction of his serum amyloid A protein (SAA) levels. Meanwhile he received a cadaveric renal transplant and maintains anakinra treatment. Despite renal failure being the most feared complication of AA amyloidosis caused by TRAPS, little data is available about safety of anti-IL-1 treatment in patients with severe kidney failure. The authors report this case of a patient on dialysis treated with anakinra in which no complications were registered. Though amyloidosis is established, the authors believe containing its progression and reducing inflammatory activity can improve patient prognosis and reduce recurrence of amyloidosis in kidney transplant, as has been demonstrated in transplanted patients due to familial Mediterranean fever amyloidosis.

## 1. Introduction

Periodic fever associated syndromes are monogenic autoinflammatory diseases with autosomal patterns of genetic transmission. These hereditary febrile syndromes lack features of adaptive immune dysregulation and have been proposed to be caused by innate immune defects. Innate immune system dysregulation causes a pathogenic proinflammatory state driven by inflammasome activation and consequently excessive production of inflammatory cytokines, namely, interleukin-1-beta [1–3].

Tumor necrosis factor receptor associated periodic syndrome (TRAPS) caused by mutation of the TNF receptor superfamily 1A gene was first characterized in 1999, though the disease had been previously described as familial Hibernian fever in 1982 [4]. Though the disease has no cure, in the last decade IL-1 receptor antagonists have emerged as the first-line treatment option for TRAPS patients [5, 6], being effective in both acute clinical symptom control and postponing of the appearance of amyloidotic complications [1, 6, 7]. Though in the past anti-IL-1 drugs were thought to be reductant when amyloid deposition had already caused significant damage to vital organs, more recent evidence points out that these agents are effective in controlling inflammation, containing amyloidosis, and improving quality of life and possibly some regression of amyloidosis could occur [7–9]. Also a great benefit in cardiovascular protection could derive from controlling inflammation itself [10, 11].

Late diagnosis of TRAPS can entail serious complications in terms of prognosis and pose difficulties for treatment. Despite renal failure being the most feared complication of TRAPS, little data is available about safety of anti-IL-1 treatment in patients with severe kidney failure.

## 2. Case Presentation

We report the case of a 44-year-old male referred by the family doctor to our nephrology clinic due to the uncontrolled hypertension and renal failure. The patient had a 3-year history of hypertension and hypercholesterolemia and he was taking the following medications: propranolol 40 mg, simvastatin 20 mg, losartan 50 mg, and nifedipine 60 mg.

This patient presented to our outpatient clinic with uncontrolled hypertension (195/110 mmHg), leg edemas that extended to the lower thighs, and complaints of fatigue and headaches. On this first consultation, the patient provided blood and urine workup from 3 months earlier that documented normocytic, normochromic anemia (Hb 12 g/dL), an elevation of creatinine and BUN to 1.9 mg/dL and 66 mg/dL, respectively, and proteinuria of 4.0 g/24 hours. He also had a renal ultrasound reporting normal sized, normal contoured, hyperechogenic kidneys bilaterally with slight corticomedullary dedifferentiation.

The patient was hospitalized with the diagnosis of nephrotic syndrome. The initial workup included a 24-hour urine collection with total proteinuria of 7.36 g, a urinary sediment with many hyaline casts, and a complete blood workup that revealed serum creatinine 2.5 mg/dL, a PTH level of 82 pg/mL, and a slight prolongation of prothrombin time. Subsequent full laboratory and imaging screening tests ruled out infectious and neoplastic or autoimmune disorders. The only positive finding was a left ventricular and auricular hypertrophy with a normal systolic function and ejection fraction, seen on echocardiogram.

From his personal medical history, he had been hospitalized on three occasions, twice in a surgical department for an appendectomy and a cholecystectomy and once in a cardiology department due to suspicion of rheumatic fever that was never confirmed. Apart from the family doctor he denied any other regular medical follow-up. He denied smoking, drugs, or excessive alcohol intake. He also denied contact with animals, except for his dog, and had never travelled abroad. From his family history, there was a history of hypertension from his father and breast cancer from his mother and one younger sister that is healthy. He had no knowledge of consanguinity or congenital diseases within his family.

During the 2-week period the patient was hospitalized, a rapidly progressive worsening of kidney function was observed with creatinine levels reaching 6.3 mg/dL accompanied by serious anemia and an altered coagulation function test needing transfusional support. The prothrombin time was not corrected with fresh frozen plasma and in need of performing a kidney biopsy, we opted for starting the patient on corticosteroids with oral prednisolone 1 mg/kg.

On the 15th day of hospitalization, the hematologic disturbances stabilized, hypertension was controlled, and a kidney biopsy was performed. Over the next days, under corticosteroids the patient's clinical status improved, with concurrent stabilization of kidney function and the anemia, to creatinine level 2.80 mg/dL and hemoglobin 11.8 g/dL. Diet modifications, antihypertensive medication, and chelators of potassium and phosphorus as well as iron therapy had been introduced, with good response, and the patient was discharged on this medication. He waited for the kidney biopsy results as an outpatient and was summoned up for consultation and reevaluation at our nephrology clinic as soon as we had the results.

The kidney biopsy revealed complete sclerosis of the glomeruli with invasion of mesangium and capillaries by an amorphous substance, with 2 glomeruli presenting hyaline mesangial proliferation. Tubules were completely atrophic and there was diffuse interstitial fibrosis with a moderate lymphocitary infiltrate. There was arteriolar hyalinosis at the vascular poles staining positive with Congo red, with normal appearance of the remnant vessels. Immunohistochemistry was negative for complement, free light chains or immunoglobulins. Evaluation for the presence of serum amyloid A (SAA) protein was positive in both the vessels and glomeruli.

Considering the biopsy results we asked the patient to come in for a clinical interview with his wife. On this interview, the patient recalled a long-time history of febrile periods since he was 9 years old that had faded with age and therefore he did not consider relevant. He had been investigated as a child but no conclusion had been made. According to the wife, their two male children suffered from a similar kind of recurrent fevers since they were about 3 years old which had been investigated by their pediatrician but so far no diagnosis had been made. Our patient reported his febrile periods lasted approximately 1 week to 10 days, with no cyclic clockwork recurrence, sometimes attributed to stressful events but mostly unpredictable, and did not respond to classic antipyretics such as ibuprofen or paracetamol. These febrile periods were often accompanied by headaches or myalgia that was focal and migratory, but he denied pharyngitis, skin rashes or urticaria, aphthous ulcers, abdominal pain, arthritis, or vomiting. His children had febrile periods with somewhat different characteristics, as they lasted longer, usually 2 weeks, had a cyclic recurrence 6 months apart, and were mostly accompanied by abdominal pain and migratory arthritis. Neither the patient nor his children had any dysmorphology. His children had normal psychomotor development for their ages, 6 and 8 years old, respectively. The whole family was tested for serum amyloid A protein (SAA). The father was positive (90.3 mg/L, normal <6.4) and the children were negative.

Considering such family history and the fever recurrence in the absence of any suspected infection, the diagnostic algorithm of periodic fever syndrome was considered and corticosteroid therapy was gradually tapered. The fact that both children were affected made us focus on disorders with an autosomal dominant pattern of transmission. Though the father and children had some phenotypic variability, the febrile periods were > 1 week long in all of them, they had consistently 2 to 3 episodes a year, and they all lacked lymphadenopathies and cutaneous or ophthalmologic manifestations, which made us think of TRAPS as the most probable diagnosis.

The family was referred to a geneticist and was tested for TNF receptor mutations. All subjects were positive in heterozygosity for a missense variant mutation (c.242G>T p.Cys81Phe) of the TNFRSF1A gene on chromosome 12, and the diagnosis of TRAPS was confirmed. When these results were known, the family was referred to the rheumatology department to be considered for treatment with IL-1 receptor antagonists. The children were started on anakinra and up until the moment of submission of this article, the children have had no complications of treatment and they have not had any febrile episode for 2 years.

In the meanwhile, the patient continued follow-up in our nephrology clinic and had the need for hospitalization twice, 11 and 13 months after the first hospitalization, due to recurrent pericarditis that did not respond to NSAIDs nor corticosteroid therapy but did respond partially to colchicine. Approval for the use of anakinra in our patient took longer, as anakinra is not approved for use in patients with severe renal failure. Despite control of hypertension and dyslipidemia and partial remission of proteinuria, the patient had a protracted course into end-stage renal failure starting hemodialysis 15 months after the first hospitalization. While on hemodialysis the patient was started on anakinra (100 mg every two days, subcutaneously). Throughout treatment the patient had no serious complication and remained symptom-free, with marked reduction of his serum amyloid A protein levels (3.3 mg/L).

The patient received a successful cadaveric renal transplant in December 2017, 15 months after starting dialysis, and maintains treatment with anakinra until today, which was adjusted after kidney transplantation to 100 mg daily, subcutaneously.

## 3. Discussion

Periodic fever associated syndromes are monogenic autoinflammatory diseases with autosomal patterns of genetic transmission. Among the classic periodic fever syndromes TRAPS is generally distinguished by being characterized by longer febrile periods, with an average of 7 days to 3 weeks; however there is great interindividual variability in clinical features [2, 12]. Most typical clinical features associated with TRAPS, apart from fever, are abdominal pain (75%), headaches (68%), and focal migratory myalgia (64%) [2], though many others have been described [12–14]. There seems to be a difference on clinical manifestations between patients with pediatric and adult disease onset, with older patients tending to have longer attacks duration and presenting more frequently with chest pain and headache, while abdominal pain, vomiting, cervical adenitis, and pharyngitis predominate in pediatric patients [14]. Recurrent pericarditis is common, especially when disease onset is in adulthood and is more frequently associated with low penetrance mutations, though it was not this patient's case [15, 16].

To date there are more than 170 sequence variant mutations of the TNFRSF1A gene listed on ClinVar [17], though the exact clinical significance of some mutations is unknown. Mutations affecting certain cysteine residues of the TNF receptor seem to be associated with more serious disease and higher risk of systemic amyloidosis, though there is not an exact correlation between genetic mutation and clinical phenotype. Missense mutations, like the mutation our family has, are nonconservative amino acid substitutions, which is likely to impact secondary protein structure and consequently its function [5, 18, 19]. Missense variants in the cysteine residues (C81G/R/Y) and in nearby residues (T79M/K, C84R/Y/S, E85D) have been reported in the Human Gene Mutation Database in association with periodic fever [20]. The largest series of patients with TNFRSF1A gene mutations, EUROFEVER/EUROTRAPS, reported that pathogenic mutations affecting cysteine residues occurred in about 27% of TRAPS patients [2]. Notably, patients with such mutations had a 25% risk of developing secondary amyloidosis, in contrast to patients with low penetrance mutations (R92Q, P46L) which have a 2% risk of developing systemic amyloidosis [1, 2, 7]. The exact mechanism by which a mutation of the TNF receptor leads to activation of inflammatory response is not completely understood, probably because not all mutations share the same mechanism of disease. Some variants of the TNF receptor mutation course with decreased levels of the soluble TNF receptor which lead to insufficient neutralization of excess TNF*α*. These variants often respond to etanercept, an anti-TNF*α* monoclonal antibody [8]. However, some genetic variants seem to produce misfolded TNF receptors which are retained in the endoplasmic reticulum and act as sensitizers to small stimuli from the innate immune system, causing the cells to have an exaggerated inflammatory response from trivial stimuli [9]. Consistent with this last theory, called the “misfolding hypothesis,” is the demonstration that cysteine residues are involved in formation of intramolecular disulfide bonds, essential to maintain the three-dimensional structure of TNFR1 [10].

Primary treatment of TRAPS targets symptom control and secondary treatment focuses on prevention of long-term complications, namely, AA amyloidosis. Acute symptom control was classically treated with colchicine and oral corticosteroids, with variable results. In the last decade IL-1 receptor antagonists have emerged as the first-line treatment option for TRAPS patients [20–26], being effective in both acute and long-term treatment. Currently 3 anti-IL-1 drugs are available; all of them are renally excreted and caution is advised for use in patients with renal failure. Anakinra, a recombinant homolog of the human IL-1 receptor that competitively inhibits binding of IL-1-alpha and IL-1-beta to its receptor, was the first of its class and has the handicap of needing daily subcutaneous administration with frequent skin reactions. Long-lasting drugs targeting IL-1-beta, such as canakinumab and rilonacept, have better tolerability [11, 27] and are administered subcutaneously every 4 to 6 weeks; however trials evaluating their safety profile in renal failure are lacking. Data regarding the use of IL-1 receptor antagonists in patients with end-stage renal failure is limited to case reports of single patients [28–30], both using anakinra.

Current evidence points to anti-IL-1 drugs being effective, even when significant organ damage has occurred, by controlling inflammation and containing further amyloid deposition. Apart from symptomatic control with anakinra our patient was offered a cadaveric renal transplantation, both of which greatly improved his quality of life. The patient should maintain anakinra throughout his life to prevent long-term complications of this autoinflammatory disorder. Whether this drug will be absolutely effective in containing amyloid deposition in the renal allograft should be a matter for future studies.

## Abbreviations

IL-1: Interleukin-1-beta
SAA: Serum amyloid A protein
TNF: Tumor necrosis factor
TNFR1: Tumor necrosis factor receptor 1
TRAPS: Tumor necrosis factor receptor associated periodic fever syndrome.
